# Risk Assessment of Infection by Airborne Droplets and Aerosols at Different Levels of Cardiovascular Activity

**DOI:** 10.1007/s11831-021-09613-7

**Published:** 2021-07-01

**Authors:** Jana Wedel, Paul Steinmann, Mitja Štrakl, Matjaž Hriberšek, Jure Ravnik

**Affiliations:** 1 Institute of Applied Mechanics, University of Erlangen-Nuremberg, Erlangen, Germany; 2 Glasgow Computational Engineering Center, University of Glasgow, Glasgow, UK; 3 Faculty of Mechanical Engineering, University of Maribor, Maribor, Slovenia

## Abstract

Since end of 2019 the COVID-19 pandemic, caused by the SARS-CoV-2 virus, is threatening humanity. Despite the fact that various scientists across the globe try to shed a light on this new respiratory disease, it is not yet fully understood. Unlike many studies on the geographical spread of the pandemic, including the study of external transmission routes, this work focuses on droplet and aerosol transport and their deposition inside the human airways. For this purpose, a digital replica of the human airways is used and particle transport under various levels of cardiovascular activity in enclosed spaces is studied by means of computational fluid dynamics. The influence of the room size, where the activity takes place, and the aerosol concentration is studied. The contribution aims to assess the risk of various levels of exercising while inhaling infectious pathogens to gain further insights in the deposition behavior of aerosols in the human airways. The size distribution of the expiratory droplets or aerosols plays a crucial role for the disease onset and progression. As the size of the expiratory droplets and aerosols differs for various exhaling scenarios, reported experimental particle size distributions are taken into account when setting up the environmental conditions. To model the aerosol deposition we employ $\text{OpenFOAM}$ by using an Euler-Lagrangian frame including Reynolds-Averaged Navier–Stokes resolved turbulent flow. Within this study, the effects of different exercise levels and thus breathing rates as well as particle size distributions and room sizes are investigated to enable new insights into the local particle deposition in the human airway and virus loads. A general observation can be made that exercising at higher levels of activity is increasing the risk to develop a severe cause of the COVID-19 disease due to the increased aerosolized volume that reaches into the lower airways, thus the knowledge of the inhaled particle dynamics in the human airways at various exercising levels provides valuable information for infection control strategies.

## Introduction

The respiratory infectious disease COVID-19, caused by “Severe Acute Respiratory Syndrome Coronavirus 2” (SARS-CoV-2) emerged as a global health threat in 2019 and is still affecting the life of humans worldwide [61]. Even in our modern world, respiratory infectious diseases can cause millions of deaths and destroy the economy leading to social instability, whose impacts remain long after the end of the pandemic [22, 61]. The transmission of the pathogens occurs mainly by three routes, the fomite, droplets and aerosol route [54, 60]. Current infection control regulations, like hand washing and $1.5\,m$ social distancing, focus most on fomite and droplet transmissions and are labeled “droplet precautions”, which are effective for diseases thought to spread predominantly by larger droplets [33]. Recently, the scientific focus shifted to the aerosol transmission route and the necessity for “airborne precautions”, due to the ability of small aerosols particles to linger prolongedly in the air [8, 33]. The SARS-CoV-2 viruses can potentially spread over large distances via the aerosol route as they remain viable in air up to $3\,h$ and thus undermine the effect of distancing regulations [8]. Therefore, contagious aerosols could increase the risk of infection in a largely extended neighborhood [37]. For SARS-CoV-2 the virus can bind and enter the host cell with the receptor angiotensin-converting enzyme 2 (ACE2), which is highly present in sites of the pulmonary alveoli, a region of presumptive severe disease development [41].

The size of the contagious expiratory droplets is crucial for the disease onset and progression [8, 56]. Large droplets ($d_p > 50\, \upmu \text{m}$) are predominantly affected by gravity and follow a ballistic trajectory [33]. They can impact with surfaces or settle to the floor within a meter of the source [33]. Droplets with $10\,\upmu \text{m} \le d_p \le 50\, \upmu \text{m}$ can reach further as they can be carried by the air flow for more than $2\,m$ [33]. Droplets with $d_p<10\,\upmu \text{m}$ can travel long distances via air currents as they are much less prone for settling [33]. Moreover, small droplets and aerosols can bypass the mechanical lung defense mechanisms and therefore penetrate deep in the airway to the alveolar region, whereas larger droplets tend to deposit mostly in the upper airways [8, 56, 57]. Despite the fact that an infected person with SARS-CoV-2 predominantly experiences symptoms of mild upper respiratory tract infection, there are cases where infections of the lower airways result in severe pneumonia, potentially leading to respiratory distress syndrome (ARDS) and death [39]. However, the emergence of typical lung disease profiles related to the alveolar region caused by SARS-CoV-2 requires that the contagious aerosols reaches the lower airways, which is a major site of disease morbidity [37, 39, 56].

Therefore, a strong interest lies in properly estimating the size distribution of exhaled droplets. It is common knowledge that expiratory events, such as sneezing, coughing, talking, and breathing, can release infectious particles [17, 32, 45]. For the size distribution of exhaled droplets and aerosols substantial literature is available for different expiratory activities. However, there is no universal particle size distribution or amount of released droplets for a specific respiratory activity as the results vary between individuals by orders of magnitude [2]. Many researchers have focused on violent expiratory events like coughing [14, 30, 31, 33, 34] and sneezing [14, 22] that yield predominantly droplets with $d_p\ge 50\,\upmu \text{m}$. The aerosols and droplets produced during sneezing and coughing are reported to vary greatly from host to host and cover a broad size range [20, 42]. However, smaller particles emitted during coughing and sneezing as well as less violent and more regular occurring actions like breathing [15, 45] and talking [7, 62] are potentially likewise infectious for some diseases. The two latter actions typically generate particles that have a predominant diameter of $d_p\le 1\upmu \text{m}$ [17]. The most probable mechanism to explain generation of small contagious particles in breathing is a bursting mechanism of the mucus fluid-film within the bronchioles, but there are other theories such as vocal cord closure and vibration in the larynx [1, 18]. These mechanisms might explain the spreading of COVID-19 via asymptotic hosts [49]. Despite being often underestimated, speaking can release significantly larger numbers of droplets compared to coughing, as reported in early works by Papineni and Rosenthal [45] as well as Loudon and Roberts [36]. They observed that, while counting aloud, up to 10 times as many particles than in one single cough were released. Moreover, Loudon and Roberts [36] investigated the risk of singing and showed that six times more airborne droplet nuclei were emitted by singing compared to coughing. More recent work have supported these findings. Chao et al. [7] stated that counting aloud released more than six times as many droplets as a single cough. Lindsley et al. [34] detected slightly more influenza virus in cough generated aerosol than in exhalation aerosol particles. However, they stated that breathing may generate more airborne infectious material than coughing over time [34]. A comprehensive summary of experimentally obtained particle size distributions including method descriptions is provided in [22].

Through global efforts of COVID-19 researchers, effective vaccines have already been developed and are now gradually being made available to the general public. Despite the continuing high risk of infection due to viral mutations and associated uncertainties, a demand for loosening of regulations emerges among the population. This rises the need to properly analyse the risk of various activities in order to properly adjust infection control planning. While huge efforts have been made from scientists across the globe to understand the spreading of COVID-19 as well as the cellular entry of SARS-CoV-2, much less attention is paid to how potentially contagious droplets and aerosols deposit in the respiratory system during different activities. Therefore, the aim of the present study is to characterize the deposition distribution of aerosolized droplet volume in the airways while exercising upon exposure to different particle size distributions covering a range of respiratory activities like sneezing, coughing and breathing. In this study, the risk of various exercise levels is evaluated from a fluid mechanical point of view where the question is targeted, whether higher level exercising renders a decisive difference in aerosol deposition behavior and consequently virus load compared to resting. Regarding the fact that in vivo experiments are limited due to human safety, computational fluid dynamics (CFD) can be employed to provide new insights in this field. In this paper we employ a numerical lung model setup which has already been successfully validated by Wedel et al. [57] in comparison to the in vitro and in silico benchmark case of Koullapsis et al. [29].

In this study we conjecture a correlation between the number of transported viruses to each lung region and the local amount of deposited saliva volume. In this context, we associate a rising risk of developing a severe cause of the disease with an increasing number of pathogens penetrating into the deeper lung regions. In addition, it should be noted that this study employs a simplified human respiratory system that is limited to oral inhalation as the nasal cavity is omitted.

The paper is organized as follows: In Sect. 2, pulmonary ventilation is introduced. Moreover, Sect. 3 describes the modeling of expiratory particles. In addition, Sect. 4 reviews the governing equations. Furthermore, Sect. 5 contains the computational setup of the employed lung model as well as the resulting flow fields for various exercising levels. In addition, Sect. 6 contains local volumetric aerosol deposition across varying exercise levels for three different scenarios. Finally, Sect. 7 summarizes the paper and presents the main conclusions.

## Pulmonary Ventilation

One main function of the lung is to enable gas exchange between the circulatory system and the external environment [46]. The lungs are composed of branching airways that end up in respiratory bronchioles and the alveolar region, which participate in the gas exchange [46]. The inspired or expired volume of air per minute is denoted as ${\dot{V}}_e$ and is referred to as minute ventilation [27]. It is the product of an average breathing frequency ${\bar{f}}_B$ per minute and an average tidal volume ${\bar{V}}_T$:

1 $$\begin{aligned} {\dot{V}}_e={\bar{f}}_B {\bar{V}}_T. \end{aligned}$$

The average ventilatory parameters of a resting adult is a breathing frequency, also referred to as respiratory rate, of ${\bar{f}}_B=12$ breaths/min and a tidal volume of ${\bar{V}}_T=500$ ml rendering a minute ventilation of ${\dot{V}}_e=6$ l/min [27]. During vigorous exercising the minute ventilation may rise up to 180   l/min depending on the athlete and type of sport [27]. With exercise ${\dot{V}}_e$ is increased as a direct function of the oxygen needed at the cell level and the carbon dioxide produced by the muscles, which is achieved with an increase in ${\bar{f}}_B$ or ${\bar{V}}_T$ or both [27]. During progressive exercise ${\dot{V}}_e$ rises through increase in ${\bar{V}}_T$ and ${\bar{f}}_B$ [27]. However, at high levels of exercise further increases in ${\dot{V}}_e$ is predominantly achieved through ${\bar{f}}_B$ whereas ${\bar{V}}_T$ reaches a plateau [27]. In Table 1 we provide an overview of typical minute ventilation rates for various types of exercises.

**Table 1:** Minute ventilation (${\dot{V}}_e$) of various exercising levels

${{\dot{V}}_e}$ [l/min]	*Re* [–]	Potential activity	Exercising level	Sources
$\approx 6^{\mathrm{a}}$	416	Resting, Sleeping	Rest	[12, 27, 50]
12	833	Driving Car, Driving Bus	Low	[38]
25	1735	Driving Bicycle	Low	[38]
50	3469	Driving Bicycle	Moderate	[38]
75	5204	Runners and cyclists	Moderate	[44]
100	6938	Runners and cyclists	Moderate	[44]
125	8673	Runners and cyclists	Vigorous	[44]
150	10,407	Runners and cyclists	Vigorous	[44]
		Soccer player at peak exercise	Vigorous	[12]

$${}^{\mathrm{a}}$$ $${\bar{V}}_T=0.5\,l$$, $${\bar{f}}_B=12$$  breaths/min

## Aerosol Modeling

As mentioned in Sect. 1, the behavior of aerosols is largely characterized by the particle size distribution. A universal measure of expiratory particles does not exist, and the results depend strongly on the employed methodology and technology, the subjects, their health state and respiratory activity [23, 63]. Therefore a wide range of particle size distributions can be found in the literature. Respiratory particles are usually measured by their number or mass concentration [23]. By employing number concentrations of particles, the tiny particles get emphasized, whereas mass concentrations are biased towards larger particles [23].

**Table 2:** Typical size distributions of expiratory droplets and aerosols [22]

Author	Year	Method/technology	Subj.	Action	Results
Duguid [14]	1946	Solid impaction	$1^{\mathrm{a}}$	Cough&	Size range: 1–2000 $\upmu \text{m}$,
		(celluloid-surfaced		Sneeze	$95\,\%$ between 2–100 $\upmu \text{m}$;
		slide)			Droplet nuclei:
					0.25–10 $\upmu \text{m}$ (sneeze)
Loudon&	1967	Solid impaction	$3^{\mathrm{a}}$	Cough&	Geometric mean:
Roberts [36]		(chamber with		Speech	$55.5\,\upmu \text{m}$ (cough)
		bond paper)			& $85\,\upmu \text{m}$ (speech)
Papineni&	1997	Solid impaction	$5^{\mathrm{a}}$	Cough	$85\,\%$ of the particles had
Rosenthal [45]		(glass slides)			diameters of $d_p \le 1\,\upmu \text{m}$
		& opt. technology			
		(opt. part. counter)			
Edwards	2004	Optical technology	$12^{\mathrm{b}}$	Breath	Size range:
et al. [15]		(opt. part. counter)			0.15–0.19 $\upmu \text{m}$
Xi et al. [62]	2009	Solid impaction	$7^{\mathrm{a}}$	Cough&	Average size:
		(glass slides with		Speech	5–100 $\upmu \text{m}$
		microscopy)&			
		opt. technology			
		(dust monitor)			
Chao et al. [7]	2009	Interferometric Mie	$11^{\mathrm{a}}$	Cough&	Geometric mean:
		imaging technique		Speech	$13.5\,\upmu \text{m}$ (cough)
					& $16\,\upmu \text{m}$ (speech).
Fabian et	2008	Optical technology	$12^{\mathrm{b}}$	Breath	Majority of particles detected
al. [17]		(opt. part. counter)			were $0.3\,\upmu \text{m} - 0.499\,\upmu \text{m}$

$${}^{\mathrm{a}}$$Healthy

$${}^{\mathrm{b}}$$Influenca patients

In our study the particles are considered to be expiratory aerosols with a density of $\rho _p=1704\,\text{kg/m}^3$ as proposed by Lindsley et al. [30]. To obtain reliable statistics in our simulations, we release 100,000 aerosol dropelts that are distributed randomly at the inlet. Furthermore, the droplets are considered to be at rest at the start of the inhalation. Since the particle response time is very short, they accelerate to the fluid velocity almost instantaneously. This happens at the entrance of the mouth region. The diameters of the inserted particles depend on the chosen size distribution. A variety of experimentally measured expiratory particle distributions is provided in Table 2. According to literature the airborne respiratory droplet range is 0.1–8 $\upmu \text{m}$ for healthy subjects and 0.05–10 $\upmu \text{m}$ for patients [63]. To cover a wide range of experimental results for expiratory aerosols and droplets resulting from different respiratory activities, the following particle size distributions are considered:

- Duguid [13]: Sneezing (droplet nuclei),
- Chao et al. [7]: Coughing (droplets, aerosols),
- Fabian et al. [17]: Breathing (aerosols)
- Artificial distribution: $$0.1\,\upmu \text{m}\le d_p \le 0.3\,\upmu \text{m}$$.

**Fig. 1:**
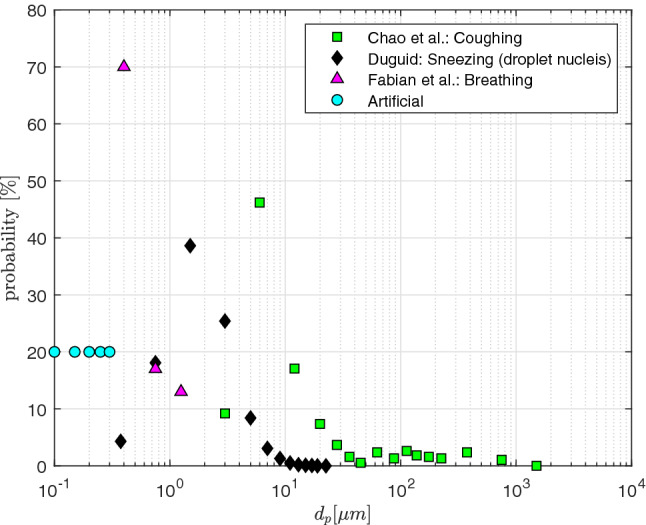
Probability of expiratory aerosol and droplet sizes

The artificial distribution is chosen to compensate for the shortfall of measuring sub-micron particles with $d_p<0.3\upmu \text{m}$ in exhaled air. By employing the different experimental and artificial particle distributions, aerosols and droplet sizes are covered in the range of 0.1–1500 $\upmu \text{m}$, which is showcased in Fig. 1. The lower aerosol size limit is set to match the approximate size of a SARS-Cov-2 virus ($d_{virus}\approx 0.1 \,\upmu \text{m})$ [4]. Due to evaporation, such aerosol sizes are possible and expected to be found in rooms some time after the air was contaminated. The mucus layer on the inner walls of the airways is mimicked by assuming that particles stick to the lung once they get into contact with the airway  [29]. Furthermore, the particle tracking time step is adjusted for each exercising level to ensure a maximum particle Courant number of $Co_p\le 1.0$.

## Methods

### Airway Geometry

**Fig. 2:**
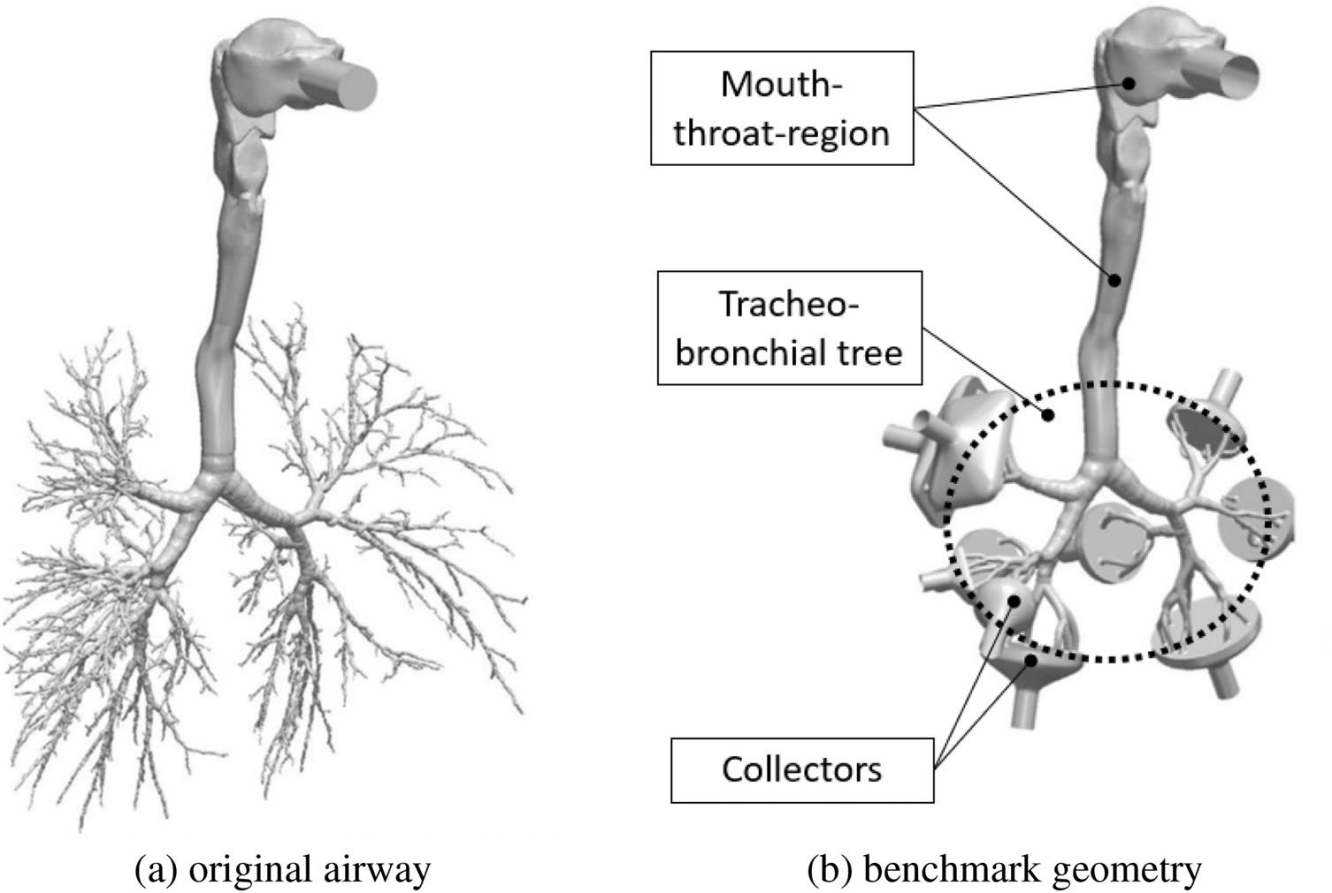
Comparison of original airway and benchmark geometry model [29]

Unlike artificial airway geometries that are often based on Weibel et al.  [58], we use a realistic, yet simplified replica obtained from medical imaging. The employed airway geometry see Fig. 2b was provided by Koullapsis et al. [29] and is originally used in [5, 6, 24, 35]. Koullapsis et al. [29] adopted this model to conduct in vitro and in silico measurement of the regional deposition ratios of di-2-ethyl hexyl sebacate (DEHS) particles. Moreover, in Wedel et al. [57] the model was employed to compare regional aerosol deposition across various age-groups.

### Governing Equations

To investigate dispersed flows of spherical particles, an Euler-Lagrangian frame is employed.

#### Flow Field

The Euler frame is used to solve the flow field inside the human airways. In conformity with Wedel et al. [57], we obtain the incompressible steady state flow in the human airways by employing the Reynolds Averaged Navier Stokes (RANS) equations in combination with the k-$\omega$-SST turbulence model using $\text{OpenFOAM}$ [43, 59]. The governing incompressible RANS equations are given by: [59]

2 $$\begin{aligned} \mathrm{d}_{\mathrm{t}}(\rho \bar{{\mathbf{u}}})+\mathrm{div}(\rho \bar{{\mathbf{u}}}\otimes \bar{{\mathbf{u}}} + \varvec{\tau }^{\mathrm{RANS}} )= -\mathrm{grad}{\bar{p}} + \mathrm{div}\bar{\varvec{\tau }} +\bar{{\mathbf{f}}}_{D} \end{aligned}$$

and

3 $$\begin{aligned} {\mathrm{div}}\bar{{\mathbf{u}}}=0. \end{aligned}$$

The Reynolds stress $\varvec{\tau }^{\mathrm{RANS}}$ and the mean viscous stress $\bar{\varvec{\tau }}$ are obtained by: [19]

4 $$\begin{aligned} \varvec{\tau }^{\mathrm{RANS}}:=\rho \bar{{\mathbf{u}}}_i' \otimes \bar{{\mathbf{u}}}_j'\, \end{aligned}$$

and

5 $$\begin{aligned} \bar{\varvec{\tau }}:=\mu \,\mathrm{grad}^{\mathrm{SYM}} \bar{{\mathbf{u}}}. \end{aligned}$$

$\text{OpenFOAM}$ uses the finite volume method (FVM) to discretise the above equations. In Eqs. 2–5$\bar{{\mathbf{u}}}$, ${\bar{p}}$ and $\rho$ denote the Reynolds-averaged fluid velocity components, the pressure and the fluid density. Additionally, the $'$-sign in Eq. 4 represents fluctuations. Body forces are captured by $\bar{{\mathbf{f}}}_D$. In order to obtain a closed system of equations and therefore constitutively express $\varvec{\tau }^{\mathrm{RANS}}$, an approximate k-$\omega$-SST turbulence model is employed [19].

#### Particles

The motion of the dispersed particles are described in the Lagrangian frame [21]. To this end, a set of ordinary differential equations, i.e. Newton’s second law, is evaluated along the particle trajectory to obtain the particle location and velocity. In $\text{OpenFOAM}$ spherical particles are treated as point masses which leads to:

6 $$\begin{aligned}&\mathrm{D}_{\mathrm{t}}{\mathbf{x}}_p:=\frac{d {\mathbf{x}}_p}{dt}={\mathbf{u}}_p, \end{aligned}$$

7 $$\begin{aligned}&\mathrm{D}_{\mathrm{t}} (m_p {\mathbf{u}}_p ):= m_p \frac{d{\mathbf{u}}_p}{dt}= \rho _p\frac{d_p^3\pi }{6} \frac{d{\mathbf{u}}_p}{dt} = \sum {\mathbf{F}}_i, \end{aligned}$$

where ${\mathbf{x}}_p$ is the position vector, ${\mathbf{u}}_p$ the velocity, $\rho _p$ the density and $d_p$ the diameter of the spherical particle. Moreover, $\sum {\mathbf{F}}_i$ accounts for the sum of forces acting on the particle [21].

In this study, we consider aerosols and droplets in the range of $0.1\,\upmu \text{m} \le d_p\le 1500\,\upmu \text{m}$. For particles with $d_p \ge 1\, \upmu \text{m}$ the major forces are the drag ${\mathbf{F}}_D$, the buoyancy ${\mathbf{F}}_B$ and the gravitational force ${\mathbf{F}}_G$ which simplifies Eq. 7 to:

8 $$\begin{aligned} m_p \frac{d {\mathbf{u}}_p}{d t} = {\mathbf{F}}_D + {\mathbf{g}} V_p\left[ \rho _p-\rho _f\right] \,, \end{aligned}$$

where $m_p$, $V_p$, $\rho _p$ are the mass, volume and density of the particle. Furthermore, $\rho _f$ denotes the fluid density and ${\mathbf{g}}$ is the gravitational acceleration. The drag force for spherical particles in $\text{OpenFOAM}$ is referred to as *sphereDrag* and is given by: [21]

9 $$\begin{aligned} {\mathbf{F}}_D = \frac{3}{4} \frac{\rho _f}{\rho _p} \frac{m_p}{d_p} \frac{C_D}{C_c} [{\mathbf{u}} -{\mathbf{u}}_p]|{\mathbf{u}} -{\mathbf{u}}_p|, \end{aligned}$$

with

10 $$\begin{aligned} C_D:= {\left\{ \begin{array}{ll} \frac{24}{\mathrm{Re}_r}[1+\mathrm{Re}_r^{2/3}/6]; &{} \mathrm{Re}_r\le 1000.\\ 0.424;&{} \mathrm{Re}_r\ge 1000. \end{array}\right. } \end{aligned}$$

and the the slip correction factor $C_c$, where the particle’s Reynolds number $\mathrm{Re}_r:=\rho _f d_p |\mathbf{u_p} -{\mathbf{u}}|/\mu$ is based on the relative velocity and particle diameter $d_p$   [9]. For the considered sub-micron droplets $0.1\, \upmu \text{m}\le d_p <1\, \upmu \text{m}$ in air, the rarefaction impact becomes obvious and slip velocity occurs at the particle surface which necessitates a slip correction [28]. This effect primarily depends on the Knudsen number *Kn* [28], which compares the molecular mean free path $\lambda$ to the particle diameter $d_p$:

11 $$\begin{aligned} Kn = \lambda /d_p . \end{aligned}$$

When the particle diameter is in the order of the gas mean free path, slip velocity at the particle surface occurs [28]. Based on Schaaf and Chambre [51], the flow regimes can be divided into four categories:

- continuum regime ($$Kn < 0.01$$),
- slip-flow regime ($0.01< Kn < 0.1$),
- transition regime ($0.1< Kn < 10$) and
- free molecular regime ($Kn > 10$).

By assuming ideal gas, $\lambda$ can be calculated as: [55]

12 $$\begin{aligned} \lambda =\frac{ k T}{\sqrt{2}\pi p d_m^2}. \end{aligned}$$

Here, *k* is Boltzmann’s constant, *T* is the temperature, *p* is the system pressure and $d_m$ is the collision diameter of molecules. As proposed by Cunningham [11] the slip correction factor is typically expressed as

13 $$\begin{aligned} C_c = 1+ A \frac{\lambda }{d_p/2} = 1+ 2 A Kn. \end{aligned}$$

The slip correction parameter *A* in Eq. 13 is as a function of *Kn* and three empirical constants $\alpha$, $\beta$ and $\gamma$:

14 $$\begin{aligned} A = \alpha +\beta \exp [-\gamma /2Kn]. \end{aligned}$$

The empirical constants depend on the gas type and particle material [11, 55]. Some typical slip correction factor expressions for different particle material at standard conditions for air are listed in [26, 55]. To model aerosol, we employ the findings of Rader [47] who obtained empirical constants for oil droplets in air. This leads to the following correction factor:

15 $$\begin{aligned} C_{c}^{Rader} = 1+ 2 Kn [1.209 + 0.441 \exp [-0.779/2Kn]]. \end{aligned}$$

In agreement with Koullapsis et al. [29] we neglect other forces like Brownian motion, added mass, and Basset history force [21].

To account for the interaction of the particles with the turbulent eddies in the RANS framework, additional models are required to approximate the fluctuation velocity. Therefore we employ the $\text{OpenFOAM}$ model *StochasticDispersionRAS* [25]. In this model a fluctuation velocity ${\mathbf{u}}'$ is computed to disturb the velocity field in a random direction, with a Gaussian distribution of zero mean and variance $\sigma \,$ [29]. This fluctuation is obtained as follows

16 $$\begin{aligned} {\mathbf{u}}'=\xi {\mathbf{d}} \sqrt{\frac{2}{3}k}\,, \end{aligned}$$

where ${\mathbf{d}}$ is a random vector and $\xi$ a random number with zero mean and unit variance of Gaussian distribution. Moreover, *k* denotes the turbulent kinetic energy [21]. A drawback of this model is the assumption of isotropic turbulence, rendering the standard deviation $\sigma$ as

17 $$\begin{aligned} \sigma =\sqrt{\frac{2}{3}k}=\sqrt{u_{1}'^2}=\sqrt{u_{2}'^2}=\sqrt{u_{3}'^2}\,, \end{aligned}$$

with $u_{1}$, $u_{2}$, $u_{3}$ denoting the velocity components in Cartesian coordinates [21, 25].

The equations are solved with the *icoUncoupledKinematicParcelFoam* solver of $\text{OpenFOAM}$.

### Limitations

The limitations of the employed set-up are in agreement with the previous study in Wedel et al. [57]:

- dilute flow allowing for one-way coupling of particles and fluid,
- assumption of isotropic turbulence and k-$\omega$-SST RANS turbulence approach,
- steady state flow field,
- considered aerosols are sufficiently small, so their surface tension is strong enough to solely behave like small spherical rigid particles [3].

In the following, the applicability of RANS turbulence approach with one-way coupling is evaluated with respect to the present investigation.

According to Elghobashi [16], the limit in particle loading which has a non-negligible influence on the flow and turbulence is at a volume fraction of $10^{-6}$. In our worst case (i.e. the smallest room and the highest volume of saliva droplets in the room) the particle volume fraction reaches only $10^{-10}$, which is well inside the limits for the validity of one-way coupling.

Crowe confirms this findings by explicitly stating “The change in turbulence intensity is correlated with the particle loading and the ratio of the particle diameter to the turbulence length scale ” [10]. The author indeed presents results where a change in turbulence intensity due to the presence of particles is shown to be a function of the ratio of the particle size $d_p$ versus the Kolmogorov length scale $\eta _K$. The change becomes substantial at $d_p/\eta _K>0.1$. The ratio of particle diameter to the Kolmogorov length scale is also examined in our earlier work [48], where a similar conclusion is reached: particles must be smaller than the Kolmogorov length scale in order for the one-way coupling point-wise approximation of particles to be appropriate. They confirm in particular, that the $d_p/\eta _K<0.1$ limit is appropriate.

The Kolmogorov length scale for a turbulent flow can be estimated by

18 $$\begin{aligned} \eta _K=\left( \frac{\nu ^3}{\epsilon _d}\right) ^{1/4}\,, \end{aligned}$$

where here $\nu =15.7\times 10^{-6}m^2/s$ is the kinematic viscosity of air and $\epsilon _d$ is the mean rate of energy dissipation. Estimating the energy dissipation via the lung diameter $d=2\times 10^{-2}\,m$ and the inlet flow velocity *u*, we obtain

19 $$\begin{aligned} \epsilon _d=\frac{u^3}{d}\qquad \Rightarrow \qquad \eta _K=Re^{-3/4}d \end{aligned}$$

and estimate the Kolmogorov length scale to be $\eta _K\approx 200\,\upmu \text{m}$ for the case of resting and sleeping ($Re=416$) and $\eta _K\approx 20\,\upmu \text{m}$ for the case of running ($Re=10407$).

To summarize, the worst case scenario in our paper considers particle volume fractions of $10^{-10}\ll 10^{-6}$, which is inside the limit for one-way coupling as proposed by Elghobashi [16], and the majority of the particle diameters considered (average sizes are $0.3\,\upmu \text{m}$ (speaking), $1.5\,\upmu \text{m}$ (coughing), $6\,\upmu \text{m}$ (sneezing)) are smaller than the Kolmogorov length scale, which according to Crowe [10] leads to the conclusion that their impact on the turbulence modulation is small. Only a small fraction of the largest particles in the analysis have dimensions comparable the the Kolmogorov length scale, but due to the extremely small particle load (volume fraction of $10^{-10}$) we conclude that it is indeed justified to neglect their impact on turbulence. Furthermore, this study targets the aerosol deposition in selected lung regions rather than precise deposition locations. Combining these statements, we consider the use of RANS with one-way coupling as appropriate in the scope of the present application. To cope with the computational-intensiveness of the present approach, reduced-order models as for example presented by Zohdi [64] are a possibility, however, supposably at the cost of accuracy.

## Flow Field at Various Exercising Levels

The simplified airway model of Koullapsis et al. [29] that is employed in this study is shown in Fig. 2b. The numerical setup and the lung mesh is identical to the model in Wedel et al. [57]. The present simulation set-up has been sufficiently validated in [57] by successfully comparing it with the benchmark results of Koullapsis et al. [29]. For convenience the numerical setup and mesh statistics of the present model are provided in Table 3. For further details refer to Wedel et al. [57] and Koullapsis et al. [29].

**Table 3:** Computational details and mesh statistics of present model

Flow solver:	RANS with k-$\omega$-SST [40]	
Inlet BC.:	*P*:	Atmospheric
	*U*:	Parabolic velocity
Outlet BC.:	*P*:	Zero-gradient
	*U*:	Specified flowrates
Mesh:	Cells	20 M
	Boundary layers	3
	Near wall distance	$y^+\approx 1$

**Fig. 3:**
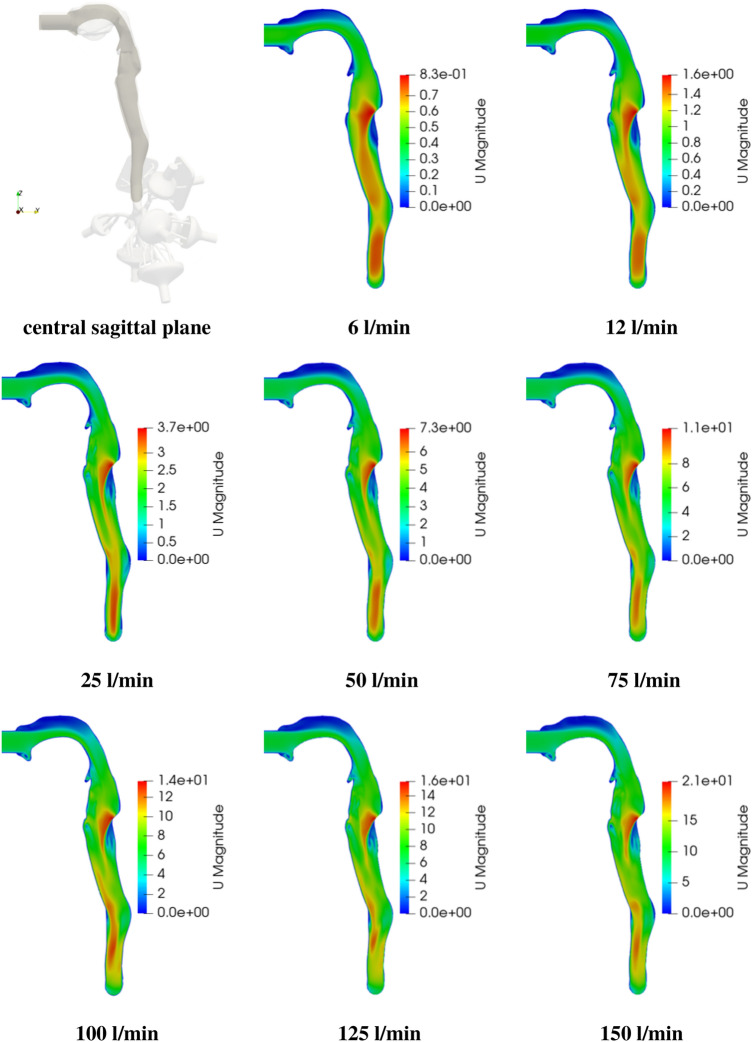
Velocity magnitude profile $|{\mathbf{u}}|$ in the central sagittal plane for various exercise levels (note the different scaling)

**Fig. 4:**
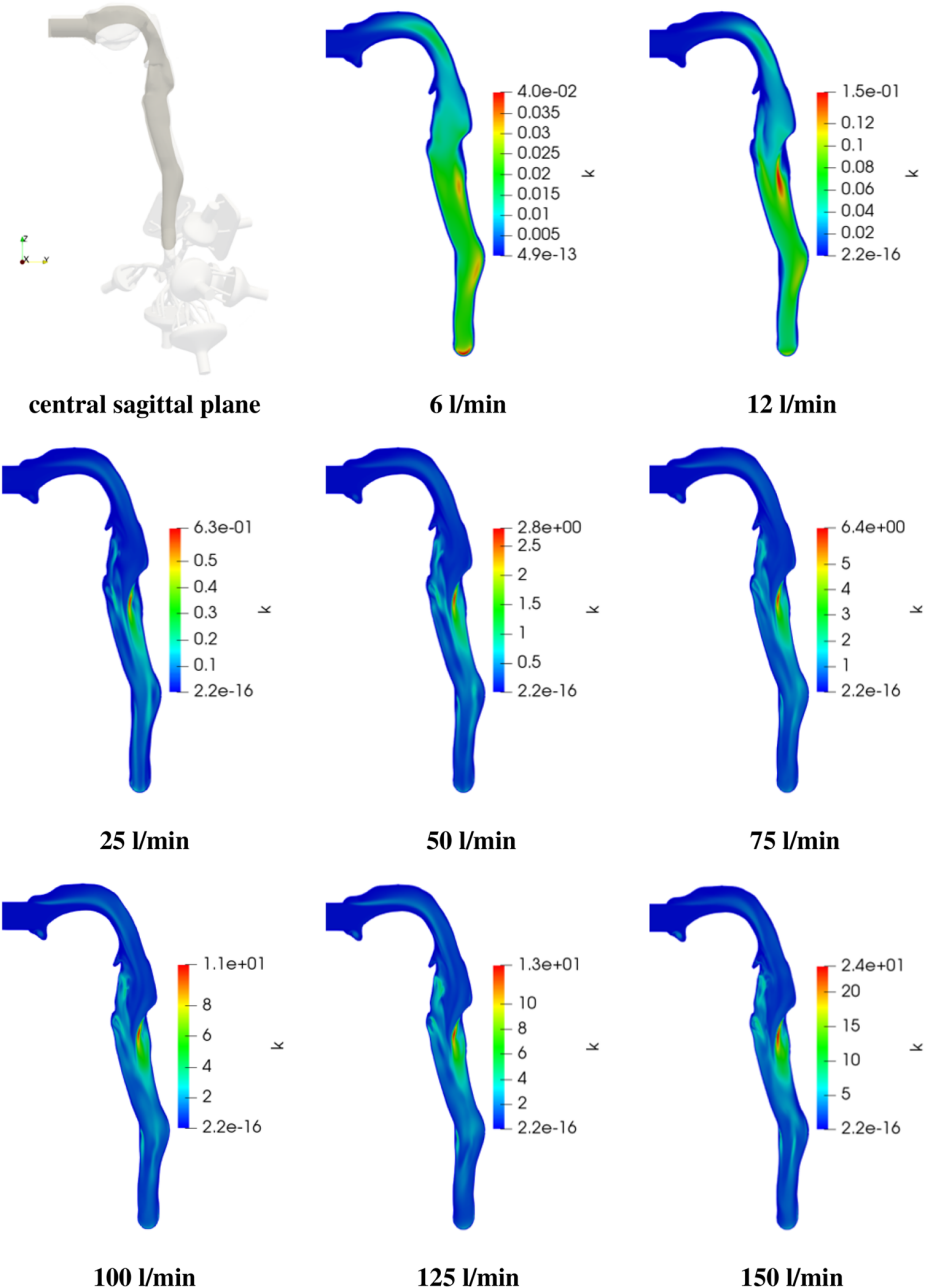
Turbulent kinetic energy *k* in the central sagittal plane for various exercise levels (note the different scaling)

As mentioned in Sect. 2, flow rates ranging from 6–150 l/min are considered, covering the resting level up to vigorous exercising. In Figs. 3 and 4 the contours of mean velocity magnitude and turbulent kinetic energy are compared in the central sagittal plane of the airways mouth-throat region for various exercising levels.

It is evident that the mean velocity distribution in the throat region is strongly varying due to changes in exercising levels. However, common feature are the velocity distribution in the oral cavity as well as the acceleration region in the back of the throat. Overall, the velocity in the central sagittal plane is strongly increasing with exercising level due to the higher flow rate. The peak velocity occurs for all exercising intensities at the beginning of the trachea in the back-throat region. Here an increase from 0.83 m/s at rest up to 21 m/s at maximum exercising level is observed. A qualitative difference in the velocity distributions across exercising levels is notable after the peak velocity in the trachea. After the acceleration region the velocity decreases only slightly in the low exercising levels (6–12 l/min). Towards higher exercising levels (${\dot{V}}_e >12$  l/min) the velocity decreases faster after the acceleration region and experiences a second acceleration close to the trachea ending. Additionally, further downstream a high velocity region is shifted to the front of the throat with increasing ${\dot{V}}_e$, leading to an asymmetric velocity distribution.

In the next step the turbulent kinetic energy distribution in the central sagittal plane is analyzed. It is visible that $k_{max}$ is significantly increasing with exercising level starting from 0.04 m$^2$/s at resting level up to 24 m$^2$/s for intense workout. For low exercising levels (6–12 l/min) the *k*-distribution in the trachea is changing predominantly in the oral cavity and is varying only slightly further downstream with a slight peak in the back of the trachea entry region. However, for higher exercising levels the turbulent kinetic energy changes more significantly along the trachea and is most pronounced at the back of the trachea.

**Fig. 5:**
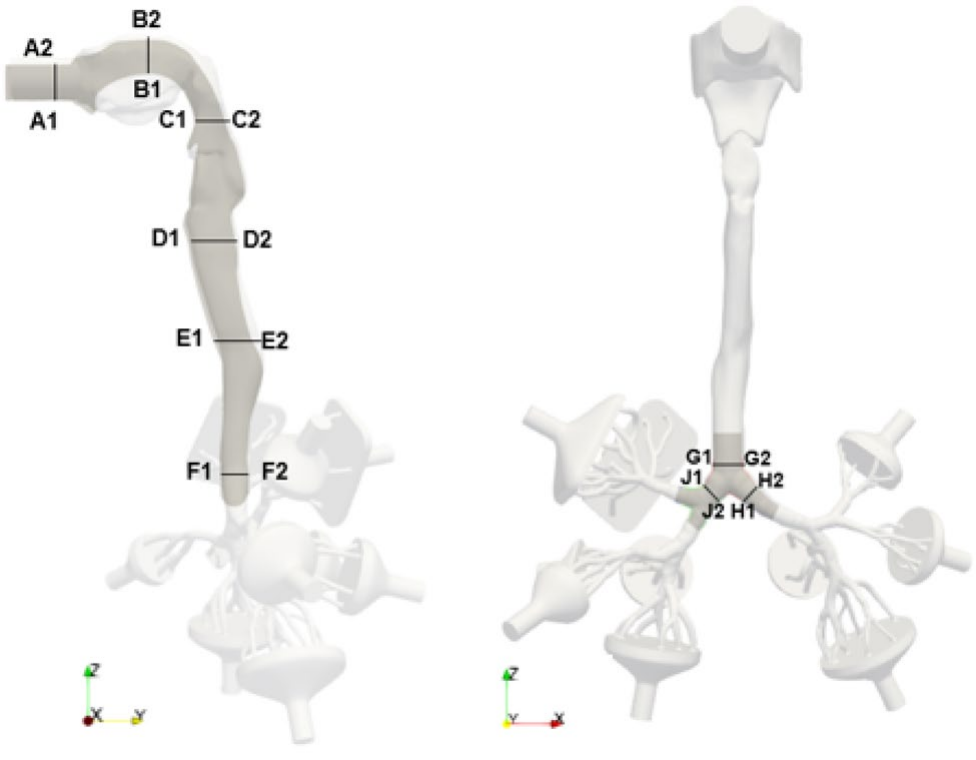
Locations of cross-sections obtained from Koullapsis et al. [29]

**Fig. 6:**
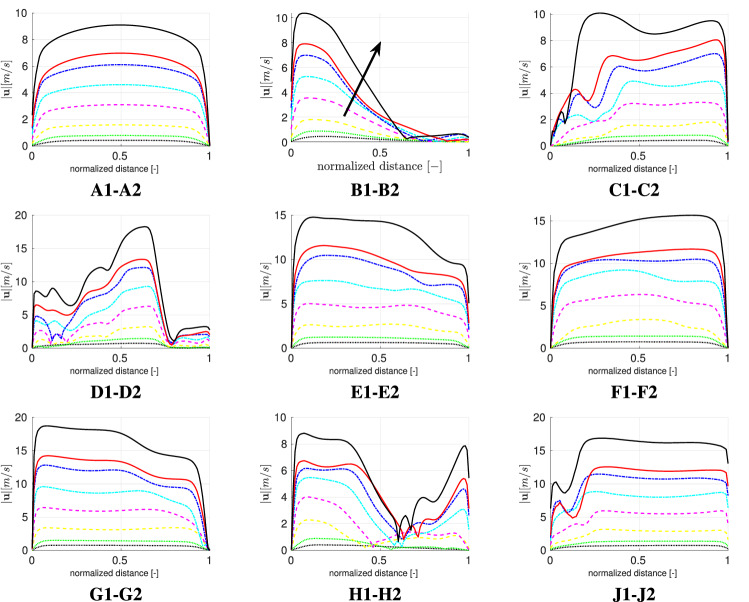
Velocity profiles for various exercising levels; 150 l/min,  125 l/min,  100 l/min,  75 l/min,  50 l/min,   25 l/min,  12 l/min,  6 l/min. Arrow indicates increasing minute ventilation

**Fig. 7:**
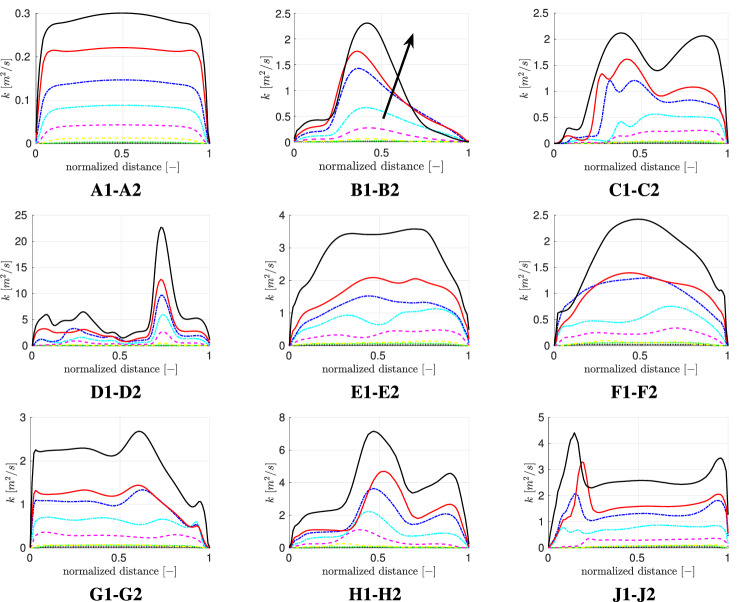
Turbulent kinetic energy profiles for various exercising levels;  150 l/min,  125 l/min,  100 l/min,  75 l/min,  50 l/min,   25 l/min,  12 l/min,  6 l/min. Arrow indicates increasing minute ventilation

In the next step a more detailed analysis of the velocity and kinematic turbulent energy fields is conducted by comparing the respective profiles at selected cross-sections. The velocity profiles are displayed in Fig. 6. Moreover, the turbulent kinetic energy profiles are displayed in Fig. 7. The location of the cross-sections are highlighted in Fig. 5 and are in agreement with Wedel et al. [57], with exact locations estimated from Koullapsis et al. [29].

Overall it is visible that the velocity magnitude is increasing with exercising level and corresponding flow rate. However, some common features can be observed. In the (A1–A2) section, which represents the inlet region, the turbulent velocity profile is visible for all exercising levels. Moreover, the typically lower velocity profiles in the low mouth depicted in (B1–B2) that is decreasing towards the back-throat, the low velocity in the pharynx region (C1–C2) as well as the acceleration region (D1–D2) are visible for all simulations. Despite the approximate similar velocity distribution in the oral cavity (A1–A2, B1–B2), a strong change in profile occurs further downstream (C1–C2, D1—D2, H1—H2, J1–J2).

For the turbulent kinetic energy, the deviation between the profiles across exercising levels gets more pronounced. Moreover, the magnitude of turbulent kinetic energy is increasing significantly in all cross-sections with increasing flow rate.

## Aerosolized Volume Deposition in Lungs Across Various Exercising Levels

In the following, expiratory droplets and aerosols are released at the inlet of the lung geometry. Particles are tracked from inhalation until they deposit or reach the collectors, i.e. penetrate in the deepest part of the lung. As infections with the SARS-CoV-2 pathogen causes varying symptoms depending on the affected region, we divide the airways in four regions of interest:

- mouth-throat region: oral caviy and trachea,
- tracheobronchial tree: airway branches,
- overall: combination of mouth-throat and tracheobronchial tree,
- collectors: representing the lower airway regions.

### Volumetric Deposition

**Fig. 8:**
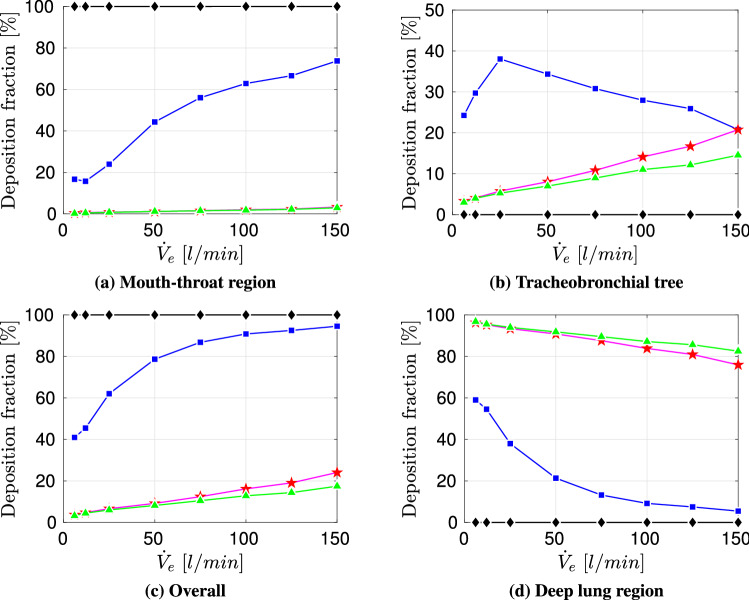
Volumetric deposition fraction of four different particle size distributions across various exercising levels; Particle size distributions:  Chao et al (coughing),  Duguid (droplet nuclei of sneezing),  Fabian et al. (breathing),  Artificial (sub-micron aerosols)

As described in Sect. 3, the particles are considered to be expiratory droplets and aerosols which are generated by a host via various respiratory activities (breathing, coughing, sneezing). Table 4 provides a detailed description of the conducted particle-insertion and tracking. In the first step, we compare the volumetric deposition fraction for 100,000 inhaled particles to obtain the deposition statistics for various exercising levels as well as particle size distributions. Fig. 8 displays the volumetric deposition behavior in the four regions of interest.

**Table 4:** Computational details of particle tracking

Time integration scheme	Implicit Euler
Forces on particles	$\text{Drag}^{\mathrm{a}}$, gravity
Wall interaction	Stick
Cunningham correction ($C_c$)	$\text{Yes}^{\mathrm{b}}$,
Turbulent dispersion	Continuous random walk
Number of particles	100,000

$$^{\mathrm{a}}$$ Drag coefficient ($$C_D$$) [53].

$$^{\mathrm{b}}$$ Rader (1990) [47]

Figure 8 displays a strong variation of deposition behavior inside human airways for the considered particle size distribution. The inhaled volume of cough-generated particles (Chao et al. [7]) that consists of significantly larger droplets than the remaining particle size distributions, deposits approximately entirely in the mouth-throat region for all considered exercising levels. This leads to a protection of the tracheobronchial tree as well as the collectors, which represent the lower airways. However, for breath-generated particles (Fabian et al. [17]) as well as the artificial distribution that both consist mainly of sub-micron particles this trend is reversed. The deposition in the mouth-throat is almost negligible leading to a majority of inhaled volume that penetrates further into the lung. Additionally, for a resting adult (${\dot{V}}_e=6$ l/min) the deposition fraction in the tracheobronchial tree is below $5\%$ enabling more than $95\%$ of the inhaled volume to reach the collectors. For the considered setup with a one-time inhalation of 100,000 particles of a breathing particle size distribution we see a reduction of deposited aerosolized volume $V_{saliva}$ in the collectors with increasing exercising level which is caused by an increasing filtration in the tracheobronchial tree. By considering the particle distribution of Duguid et al. [14], who measured the droplet nuclei generated by sneezing, an overall strong increase of particle deposition in the upper airways and therefore a decrease of deposited aerosolized volume in the collectors can be seen for increasing ${\dot{V}}_e$. For lower exercising levels (${\dot{V}}_e\le 25$) the growth of overall $V_{saliva}$ deposition is contributed by an increasing deposition in both mouth-throat and tracheobronchial tree. However, for higher ${\dot{V}}_e$ the deposition fraction in the tracheobronchial tree is decreasing which is caused by the high filtering in the mouth-throat region. Due to an overall growth of deposition in the upper airways with increasing exercise intensity, the amount of $V_{saliva}$ that penetrates to the collectors is significantly reduced.

### Scenarios

All scenarios studied in the sequel encompass a range of aerosol concentrations, room sizes and exercising levels. All scenarios consist of two parts. The first part is the expelling part where an infected symptomatic (coughing, sneezing) or asymptomatic (breathing) host stays in a specified room and conducts a defined respiratory action. Moreover, we assume that each room is sufficiently ventilated to uniformly distribute the exhaled aerosolized volume in the rooms. The second part is a susceptible person that is exposed to the generated particle concentration while exercising at various levels of intensity. Additionally, for the sake of simplicity it is assumed that the infected individual leaves the room before the exposure of the susceptible to the aerosol load starts.

#### Scenario I: Constant Aerosol Concentration

Depending on the respiratory activity of the (a)symptomatic host, various ranges of droplet concentrations can be released [63]. However, the first scenario considers a fixed exhaled saliva volume that is equal among all particle size distributions. Moreover, we assume that the exhaled $V_{saliva}$ is uniformly distributed in a room leading to room specific aerosol concentrations to mimic that a host remained therein for a sufficient amount of time. The fixed aerosol volume is set to be $V_{saliva-exh.}=9.82\times 10^{-5}\,\text{ml}$ that is thought to be released by a host via varying respiratory activities. The estimated $V_{saliva-exh.}$ would exemplarily correspond to a cough where a host coughed 10 times and released 150,000 particles/cough with an average diameter of $\bar{d_p}=5\,\upmu \text{m}$. This would correspond with the findings by Lindsley et al. [31] who measured 900 to 302,200 particles/cough for influenza patients. However, by fixing the exhaled $V_{saliva-exh.}$, the absolute numbers of particles released via varying respiratory activities, like coughing or breathing, changes due to the different particle size distributions, leading to an increased amount of particles for lower sized droplets.

With $V_{saliva-exh.}$ and the room volume $V_{room}$, the constant room droplet concentration can be obtained as follows:

20 $$\begin{aligned} C_{saliva}=V_{saliva-exh.}/V_{room}. \end{aligned}$$

Therefore, the inhaled droplet volume after a certain time $t_{inh}$ in a specific room can be estimated as:

21 $$\begin{aligned} V_{saliva}= C_{saliva}\,{\dot{V}}_e\,t_{inh} . \end{aligned}$$

**Fig. 9:**
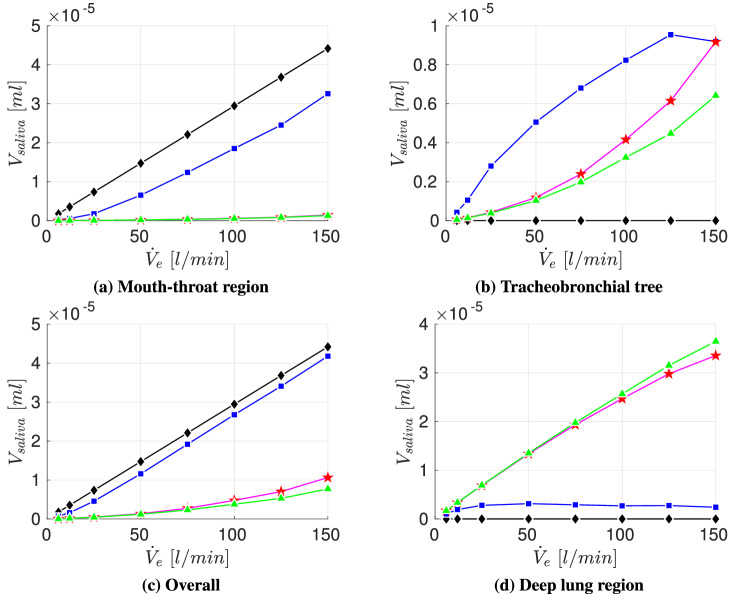
Volumetric deposition after $t_{inh}=30\,\text{min}$ of four different particle size distributions across various exercising levels for $V_{room}=10\,\text{m}^3$; Particle size distributions:  Chao et al (coughing),  Duguid (droplet nuclei of sneezing),  Fabian et al. (breathing),  Artificial (sub-micron aerosols)

In the following step the workout time is set to a typical training duration of $t_{inh}=30\,\text{min}$. Moreover, a small room with $V_{room}=10\,\text{m}^3$ is considered. The resulting deposition of inhaled $V_{saliva}$ is displayed in Fig. 9.

Figure 9 presents a clear deviation in regional aerosolized volume deposition between the considered particle size distributions. The larger particles (Chao et al., coughing) deposit entirely in the mouth-throat region with an increasing $V_{saliva}$ with intensified exercising, due to the higher amount of inhaled volume and corresponding aerosols. The breath-generated particles (Fabian et al. [17]) experience an increased volumetric deposition in the tracheobronchial tree with higher exercise level. However, the amount of $V_{saliva}$ that reaches to the collectors is likewise increasing, which is associated with an increased amount of virus that could be carried to the lower airways. In case of droplet nuclei which were generated by sneezing (Duguid et al. [14])) the deposition is increasing up to an exercising level of ${\dot{V}}_{e}=125$ l/min in the mouth-throat and tracheobronchial tree. By further intensifying the training, a decrease in deposition in the tracheobronchial tree occurs. However, the amount of $V_{saliva}$ that penetrates deep into the human lung is approximately constant for ${\dot{V}}_{e}\ge 25$ l/min.

**Fig. 10:**
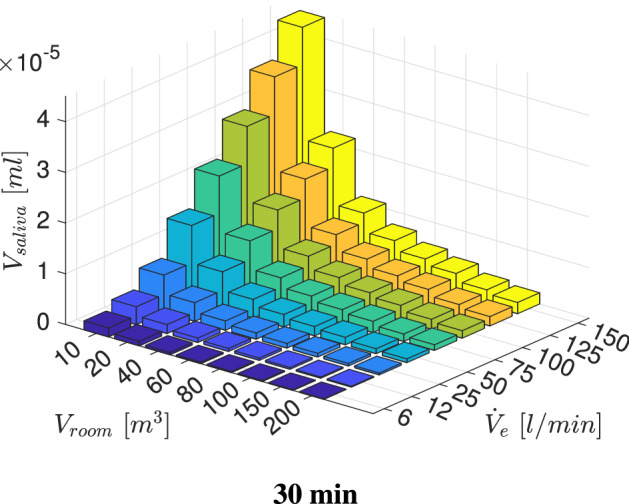
Inhaled droplet/aerosol volume after a specified time

In the next step the influence of the room size is investigated. With Eq. 21 the inhaled droplet volume for various room sizes can be estimated by employing a aerosol concentration that is constant for the particle size distributions but changing with room size. The resulting inhaled $V_{saliva}$ for $t_{inh}=30\,\text{min}$ in different room sizes across various exercise levels is visualized in Fig. 10.

**Fig. 11:**
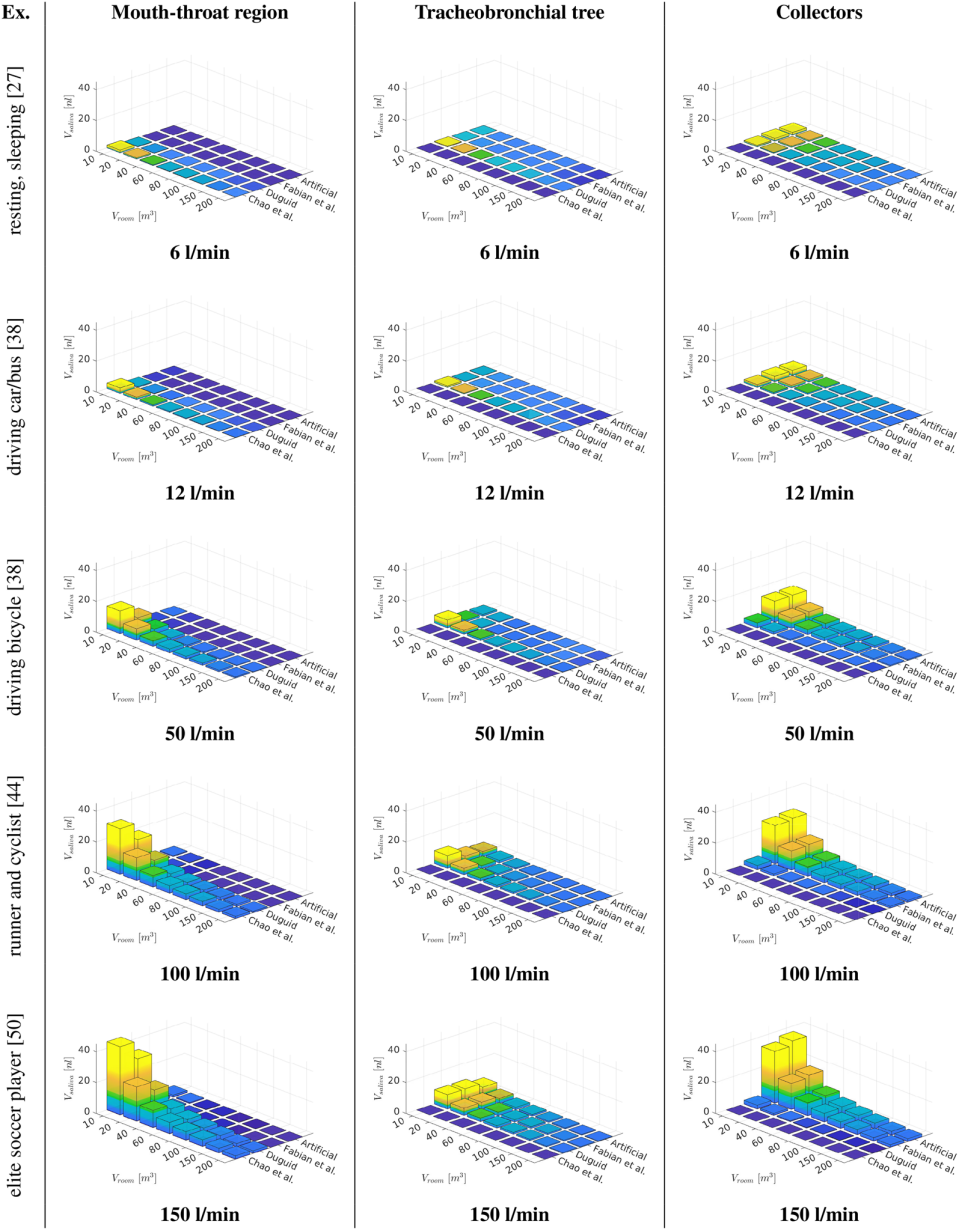
Volumetric deposition of different particle size distributions across various exercising levels (Ex.) in varying room sizes ($t_{inh}=30\,\text{min}$)

Figure 10 displays that the amount of inhaled $V_{saliva}$ is increasing with exercise level due to an increased aerosol inhalation. Likewise a shrinking in room size is entailing a growth in inhaled $V_{saliva}$ due to an underlying rise in aerosol concentration. Therefore, the maximum of inhaled $V_{saliva}$ occurs for the most intense training in the smallest room. Figure 11 presents the resulting deposition of $V_{saliva}$ for various room sizes, exercising intensities as well as particle size distributions. Across the considered exercising levels the deposition in the mouth-throat region is always highest for the smallest rooms and particle size distribution with comparably large droplets (Chao et al. [7]) with an increasing deposition towards more intense training. For lower to moderate levels of exercising the deposition is highest for small rooms and the medium sized droplet size distribution (Duguid et al. [14])) in the tracheobronchial tree. However, towards vigorous exercising with ${\dot{V}}_e=150$ l/min the deviation between medium sized particle distributions and smaller particles (Fabian et al. [17]) that could be generated by breathing is mitigated. However the volume that reaches into the collectors and therefore to the lower airways is of key interest as contagious aerosols in this region can cause typical alveolar lung disease profiles which are strongly linked to an increase in disease morbidity [56]. As displayed in Fig. 11 the amount of volume that penetrates into the lower lung is growing with intensified exercising and is significantly higher for the breath-generated particles (Fabian et al. [17]) than for sneezing droplet nuclei (Duguid et al. [14]) or cough-generated droplets (Chao et al. [7]). Despite the higher penetration to the alveolar region of small aerosols, the potential virus load can be strongly reduced by exercising in a larger room as presented in Fig. 11.

#### Scenario II: Varying Aerosol Concentration due to Respiratory Activity

In this scenario the total volume of expelled aerosolized droplets is computed according to the conducted respiratory activity of the (a)symptomatic host. Schijven et al. [52] investigated various scenarios of airborne transmission of SARS-CoV-2, obtaining expelled aerosolized volumes for coughing (mean from $3 \times 10^{-9} - 4\times 10^{-6}\,\text{ml}$) and sneezing (mean from $50\times 10^{-9} - 30\times 10^{-6}\,\text{ml}$). Exhaled breath particle size distribution and number data is obtained from Fabian et al. [17] who obtained the following particle concentration ranges in the size selective bins:

- 61–3848 particles/l (particles between $0.3\,\upmu \text{m} \le 0.5 \,\upmu \text{m})$,
- 5–2756 particles/l (particles between $0.5\,\upmu \text{m} \le 1 \,\upmu \text{m}$),
- 1–1916 particles/l (particles between $1\,\upmu \text{m} \le 5 \,\upmu \text{m}$).

With the particle size distribution and number data of Fabian et al. [17] an estimation of the lower and upper limit of exhaled aerosolized volume via breathing can be obtained by fixing a minute ventilation of 6 l/min of the (a)symptotic host and a considered time span of 30 min. This leads to an aerosolized volume in the range of $3.1\times 10^{-9} - 5\times 10^{-6}\,\text{ml}$ per 30 min. In this scenario we consider the following respiratory actions of the infected person in the specified rooms:

- one cough ($$2\times 10^{-6}\,\text{ml/cough}$$),
- one sneeze ($$15\times 10^{-6}\,\text{ml/sneeze}$$),
- breathing for 30 min ($2.5\times 10^{-6}\,\text{ml}/30\,\text{min}$).

**Fig. 12:**
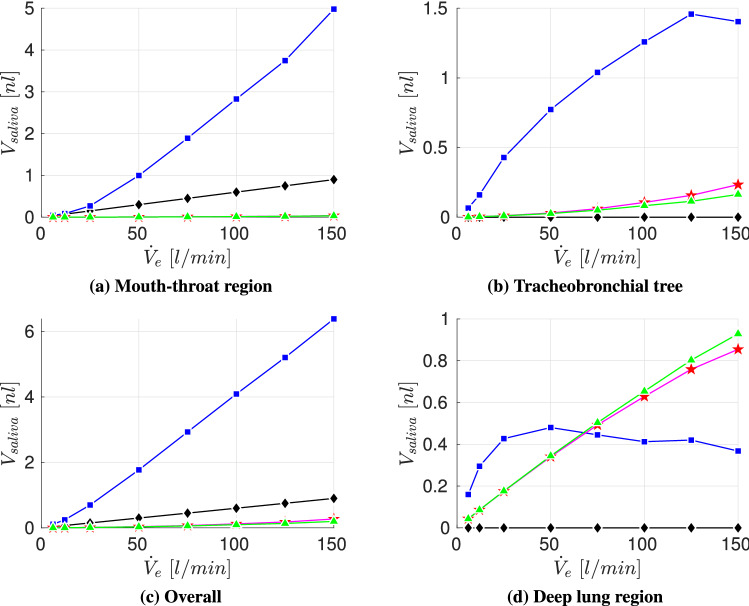
Deposited aerosolized volume in the airways after $t_{inh}=30\,\text{min}$ for four different particle size distributions across various exercising levels for $V_{room}=10\,\text{m}^3$; Particle size distributions:  Chao et al (coughing),  Duguid (droplet nuclei of sneezing),  Fabian et al. (breathing),  Artificial (sub-micron aerosols)

As displayed in Fig. 12c, the overall volumetric deposition in the airway is increasing with intensified exercising for all particle size distribution. Moreover, Fig. 12a, b presents that the saliva deposition of the particle size distribution of Duguid [14] (droplet nuclei of sneezing) is dominating in both mouth-throat and tracheobronchial tree across all exercising levels, due to the highest saliva concentration generated by a single sneeze. Despite the higher quantity of inhaled saliva, Fig. 12d displays that the amount of aerosolized volume that penetrates deep into the lung is only highest in the low to moderate exercising levels (${\dot{V}}_e < 75$  l/min). Towards more intense exercising the amount of saliva generated by a single sneeze that reaches the lower airways is decreasing. In contrary, the increase of aerosolized volume that reaches the lower lung persists towards vigorous exercising for breath generated particles (Fabian et al. [17]), see Fig. 12d). Moreover, after ${\dot{V}}_e >\,75$  l/min the breath generated saliva volume (Fabian et al. [17]) dominates over the cough generated and sneeze generated aerosolized volume in the collectors. In this scenario, breath generated particles could transport a higher amount of SARS-CoV-2 pathogens into the lower airways than the generated particles of more violent respiratory actions potentially leading to a more severe cause of the COVID-19 disease.

**Fig. 13:**
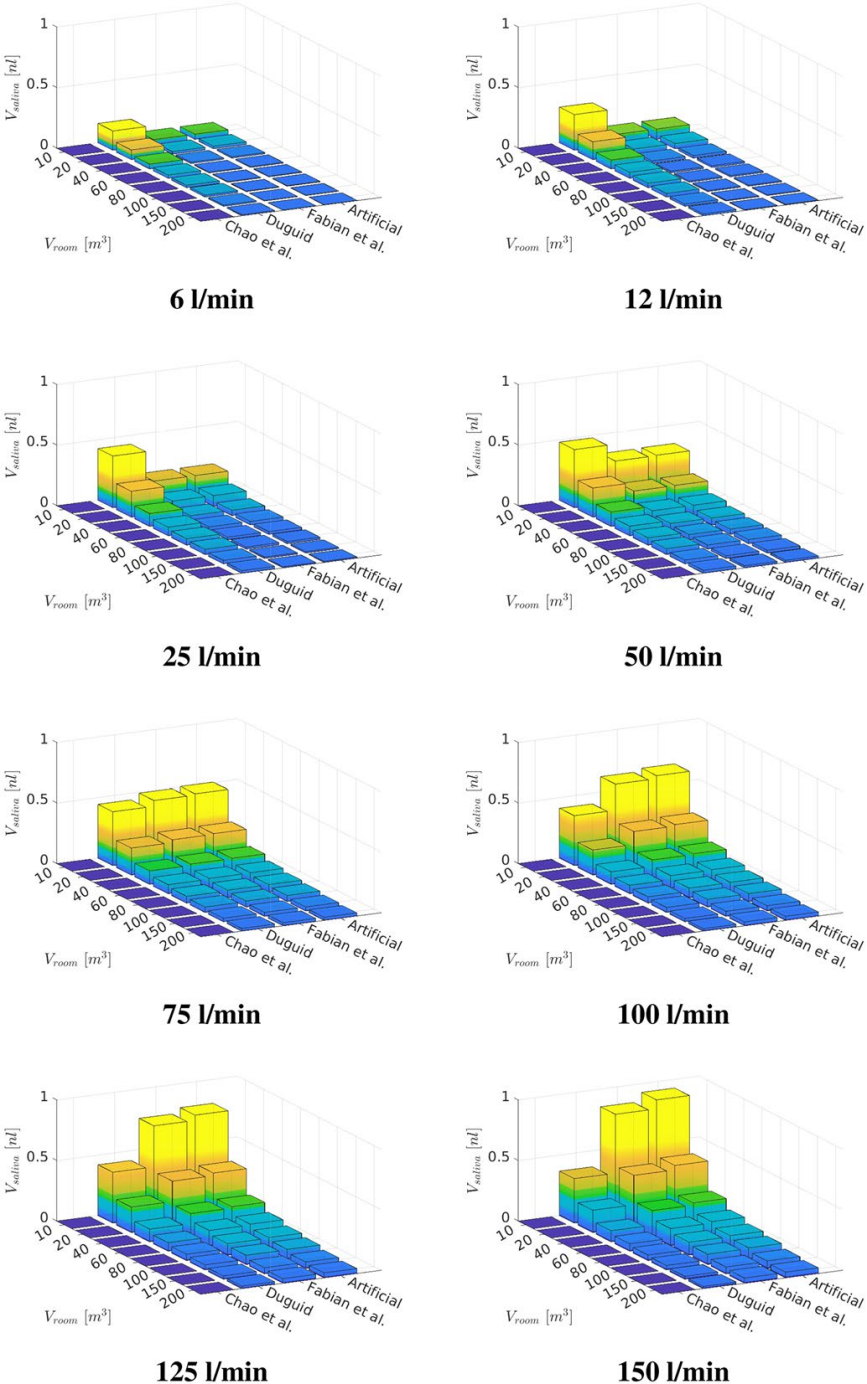
Aerosolized volume in the collectors after $t_{inh}=30\,\text{min}$ for four different particle size distributions across various exercising levels and room sizes

In the following, the volumetric deposition in the lower lung regions is compared across various room sizes. Figure 13 underlines the effect that more potentially contagious volume of the sneezing nuclei is transported to the lower airways for resting to moderate exercising ${\dot{V}}_e < 75$ l/min than by the remaining considered particle size distributions. However, with increased exercising the contribution of breath-generated droplets is dominating for all room sizes. Moreover, Fig. 13 visualizes that even if the breathing action is conducted in a double sized room compared to that of the coughing or sneezing action, breathing can still pose a higher risk as more saliva volume reaches the deeper lung region. Overall, Fig. 13 visualizes that the room size and accordingly the aerosolized volume concentration is a key factor to lower the amount of saliva volume in the collectors and therefore reduce the number of potentially harbored pathogens that could penetrate into the deep lung regions.

#### Scenario III: Comparing Asymptomatic to Symptomatic Hosts

In this scenario the total volume of expelled aerosolized droplets is computed as a combination of conducted respiratory activities. We consider the following options:

- Case A: One symptomatic host (30 min breathing + $1\, cough$ + $1\, sneeze$),
- Case B: Two asymptomatic hosts (30 min breathing).

**Fig. 14:**
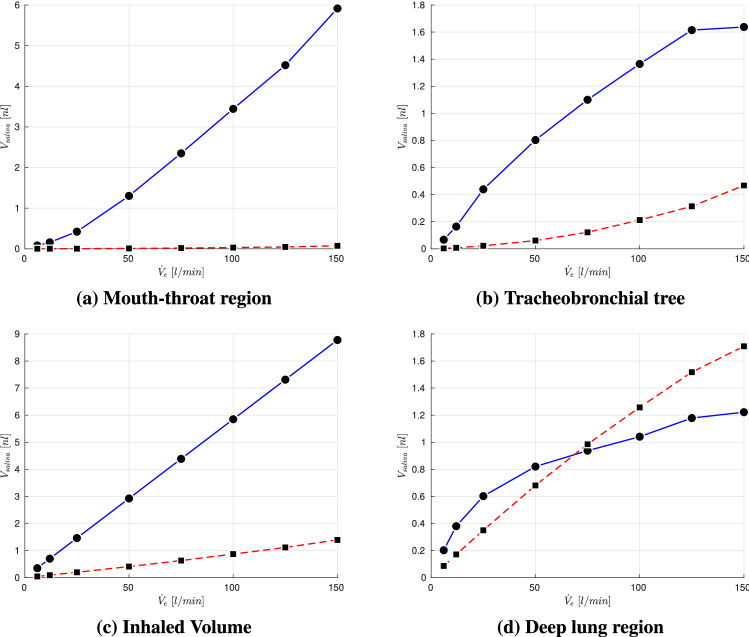
Inhaled and deposited aerosolized volume in the airways after $t_{inh}=30\,\text{min}$ for two asymptomatic or one symptomatic hosts across various exercising levels for $V_{room}=10\,\text{m}^3$; Cases:  symptomatic host,  two asymptomatic hosts

In both cases the individuals are conducting the mentioned respiratory actions and leave the specific room before a susceptible person enters. If a symptomatic host (Case A) remains in a room for 30 min, more $V_{saliva}$ is exhaled than if two asymptotic persons (Case B) were occupying an identical room for the same time. This difference is due to the larger droplet sizes generated by coughing or sneezing as compared to breathing. This leads to an increase of $V_{saliva}$ that a susceptible person would inhale in a certain time, see Fig. 14c. However, this clear trend is not directly connected to the amount of volume that penetrates to the deeper lung regions. For resting to moderate exercising, more $V_{saliva}$ reaches to the lower airways for case “A”. Despite the overall higher amount of inhaled aerosolized volume, generated by the symptomatic host (Case A), the exhaled $V_{saliva}$ of the asymptomatic hosts (Case B) poses a higher risk at more intense exercising levels ${\dot{V}}_e\ge 75$ l/min as more $V_{saliva}$ is detected in the lower airways. However, it has to be noted that for both cases the detected inhaled $V_{saliva}$ in the collectors and therefore the potential number of viruses in the alveolar region is significantly increasing with rising exercising level.

## Conclusions

In this study we employed $\text{OpenFOAM}$ to enable new insights in regional aerosol deposition in the human airway at various levels of exercising and therefore assess the infection risk of cardiovascular activities from a fluid mechanical point of view. We account for different respiratory activities of the infected host in the room, like coughing, sneezing or breathing by considering different particle size distributions from literature to establish the indoor conditions to which the exercising persons are exposed. In order to conduct this research, we employed an adult lung model which was provided by Koullapsis et al. [29]. The selected computational model which is based on the RANS equations with the k-w-SST turbulence model is identical to the model used in Wedel et al. [57] which was successfully compared to the in vitro and in silico results of Koullapsis et al. [29], rendering it as suitable for investigations of the impact of exercising levels on aerosol deposition in the human airways.

In this study we identified significant variability in regional aerosol deposition across different particle size distributions which rises the need to properly estimate the particle composition of the exhaled air of different respiratory activities. In this context, a general trend is observed, which indicates a higher deposition of saliva volume in the upper airways due to inhalation of particles that were generated by more violent respiratory actions like coughing or sneezing compared to breath-generated aerosolized volume. In addition, sneezing droplet nuclei were identified to dominate the inhaled saliva volume in the deep lung region in low to moderate exercising. However, breath generated particles are identified to pose a higher risk for the lower airway regions for vigorous exercising levels as more saliva volume is penetrating to the lower airways, which could explain the transmission through asymptomatic hosts. A higher amount of $V_{saliva}$ in the deep lung regions is associated with an increased number of viruses that could be harbored by the inhaled saliva volume. In agreement with Wedel et al. [57] we conjecture a higher virus load in the lower respiratory tract region to cause typical disease profiles of the alveolar regions (pneumonia, acute respiratory distress syndrome) which is a major site of COVID-19 morbidity. This would lead to the assumption that exercising at higher level is increasing the risk to develop a severe cause of the COVID-19 disease. However, the aerosolized volume that reaches into the lower airways can be reduced significantly by exercising in a larger room.

We conclude that a higher saliva deposition in the upper airways due to more violent respiratory activities like coughing or sneezing leads to a significant reduction of virus load in the deep lung regions, which we connect to a lower chance of infection. However, breath generated particles lead to an increasing risk of developing a severe respiratory illness originating from COVID-19 airborne transmission by intensifying the exercising level. Regarding our results, we propose to exercise in larger rooms to lower the risk of infection as it significantly reduces the amount of saliva volume that reaches into the lower airways.
